# Regulation of Two Renal Chloride Transporters, AE1 and Pendrin, by Electrolytes and Aldosterone

**DOI:** 10.1371/journal.pone.0055286

**Published:** 2013-01-31

**Authors:** Nilufar Mohebbi, Angelica Perna, Jenny van der Wijst, Helen M. Becker, Giovambattista Capasso, Carsten A. Wagner

**Affiliations:** 1 Institute of Physiology and Zurich Center for Integrative Human Physiology (ZIHP), Zurich, Switzerland; 2 Division of Nephrology, University Hospital Zurich, Zurich, Switzerland; 3 Department of Internal Medicine, Chair of Nephrology, Second University of Naples, Naples, Italy; University of Geneva, Switzerland

## Abstract

The renal handling of salt and protons and bicarbonate are intricately linked through shared transport mechanisms for sodium, chloride, protons, and bicarbonate. In the collecting duct, the regulated fine-tuning of salt and acid-base homeostasis is achieved by a series of transport proteins located in different cell types, intercalated and principal cells. Intercalated cells are considered to be of less importance for salt handling but recent evidence has suggested that the anion exchanger pendrin may participate in salt reabsorption and blood pressure regulation. Here, we examined the regulated expression of two functionally related but differentially expressed anion exchangers, AE1 and pendrin, by dietary electrolyte intake and aldosterone. Cortical expression of pendrin was regulated on mRNA and protein level. The combination of NaHCO_3_ and DOCA enhanced pendrin mRNA and protein levels, whereas DOCA or NaHCO_3_ alone had no effect. NaCl or KHCO_3_ increased pendrin mRNA, KCl decreased its mRNA abundance. On protein level, NH_4_Cl, NaCl, and KCl reduced pendrin expression, the other treatments were without effect. In contrast, AE1 mRNA or protein expression in kidney cortex was regulated by none of these treatments. In kidney medulla, NaHCO_3_/DOCA or NaHCO_3_ alone enhanced AE1 mRNA levels. AE1 protein abundance was increased by NH_4_Cl, NaHCO_3_/DOCA, and NaCl. Immunolocalization showed that during NH_4_Cl treatment the relative number of AE1 positive cells was increased and pendrin expressing cells reduced. Thus, pendrin and AE1 are differentially regulated with distinct mechanisms that separately affect mRNA and protein levels. Pendrin is regulated by acidosis and chloride intake, whereas AE1 is enhanced by acidosis, NaCl, and the combination of DOCA and NaHCO_3_.

## Introduction

The rate of renal excretion or reabsorption, respectively, of sodium and chloride determines extracellular volume and blood pressure. Stimulation of NaCl reabsorptive processes causes an expansion of extracellular volume and increases blood pressure whereas renal loss of salt is associated with low blood pressure. The importance of renal salt handling for blood pressure regulation has been highlighted by rare Mendelian syndromes with mutations in more than 20 different transport proteins, ion channels or regulators of renal salt handling [1].

Sodium and chloride may be transported by the same transport protein such as the Na^+^/K^+^/2Cl^−^ -cotransporter NKCC2 in the thick ascending limb of the loop of Henle or the Na^+^/Cl^−^ -cotransporter NCC in the distal convoluted tubule [2], [3], [4], [5]. In contrast, these ions are reabsorbed by distinct transport pathways in the late distal convoluted tubule, in the connecting tubule, and in the collecting duct [6]. Sodium is reabsorbed by the epithelial sodium channel (ENaC), which is expressed in principal cells, targeted by potassium-sparing diuretics, and regulated by a variety of hormones including angiotensin II and aldosterone [6]. Chloride appears to take two different routes, either the paracellular pathway that may be controlled by claudins [7], or the transcellular route through non-type A intercalated cells. These cells express on their luminal pole the anion exchanger pendrin (SLC26A4) reabsorbing chloride in exchange for intracellular bicarbonate in an electroneutral manner [8], [9], [10], [11], [12], [13]. The driving force for Cl^−^ -reabsorption is provided by the gradient for bicarbonate, which depends on the activity of H^+^-ATPases localized either on the luminal and/or basolateral pole of the same cells and thereby energizing pendrin-dependent transport processes [14], [15], [16]. The activity of pendrin is also indirectly regulated by angiotensin II [14], [17] and loss of pendrin reduces ENaC expression and activity linking sodium and chloride reabsorption [18], [19], [20], [21]. Moreover, mice lacking pendrin are resistant to hypertension induced by high salt and mineralocorticoid treatment [22].

A third type of cells exists along the collecting duct, type A intercalated cells, that are critical for renal acid excretion [15]. Type A intercalated cells are characterized by the expression of luminal H^+^-ATPases acidifying urine and by the basolateral presence of the chloride/bicarbonate exchanger AE1 (SLC4A1) [15], [23]. The activity of type A intercalated cells is classically not linked to blood pressure regulation. However, patients with inherited forms of distal renal tubular acidosis, syndromes of dysfunction of intercalated cells, have a mild renal salt loosing phenotype suggesting a contribution of type A intercalated cells to overall renal salt handling [24].

The response of pendrin to dietary chloride intake and distal urine chloride delivery has been studied in vivo in some detail [25], [26], [27], [28]. Promoter studies in vitro demonstrated increased transcription of pendrin stimulated by aldosterone [29] consistent with higher mRNA expression after treatment with the aldosterone analogue desoxycorticosterone acetate (DOCA) [22]. Similarly, the activity of type A intercalated cells has been reported to be stimulated by aldosterone [30], [31]. However, the impact of different electrolytes and their interactions with aldosterone has not been studied in vivo. This is of major importance to understand the regulation of these transport proteins which are critical for renal salt reabsorption and blood pressure regulation. Therefore we systematically compared the effect of various electrolytes and the additional influence of aldosterone on the expression of pendrin and AE1 in vivo in mouse kidney. Moreover, we examined the remodeling of the collecting duct by these treatments showing that mRNA and protein expression levels were affected with distinct patterns and that the relative cell numbers along the collecting duct were also modulated.

## Materials and Methods

### Animal studies and metabolic cages

Male, C57Bl/6 mice (25–30 g, Institute of Laboratory Animal Sciences, University of Zurich) were maintained on standard chow and had free access to drinking water. The mice were divided into 7 different treatment groups: 1) 0.28 M NH_4_Cl, 2) 0.28 M NaHCO_3_ and additional subcutaneous injections of deoxycorticosterone acetate (DOCA) at day 1 and 4 (2 mg/mouse), 3) 0.28 M NaCl, 4) subcutaneous injections of DOCA at day 1 and 4 (2 mg/mouse), 5) 0.28 M NaHCO_3_, 6) 0.28 M KCl, and 7) 0.28 M KHCO_3_. Each group consisted of at least 10 animals and was compared to the corresponding control group. All diets were given in drinking water in addition to 1% sucrose. Control mice were only treated with 1% sucrose. All animals were subjected to the diet for 7 days and placed in metabolic cages for the final 3 days. Daily chow and water intake, stool excretion, body weight, and urine excretion (collected under mineral oil) were monitored. At the end of the experiment, animals were perfused with PBS, and both kidneys were harvested, immediately dissected into cortex and medulla, frozen in liquid nitrogen, and stored at −80°C until further processing. Animal studies were according to Swiss Animal Welfare Laws and approved by the local Veterinary Authority (Kantonales Veterinaeramt Zurich).

### Urine analysis

Urinary pH was measured using a pH microelectrode (691 pH-meter, Metrohm, Switzerland). Urinary ammonium was determined according to the Berthelot protocol [32]. Urinary phosphate was determined by the Fiske and Subbarow colorimetric method. Briefly, standard solutions were prepared from 11.7 mM phosphate stock solution (Randox Laboratories, United Kingdom). After protein precipitation with 20% trichloroacetic acid, urine and serum samples were pipetted into a 96-well plate. After addition of acid molybdate solution and Fiske and Subbarow reducer solution (Sigma, MO, USA) the absorbance of the samples was measured at 660 nm wavelength using a μQuant microplate reader (Biotek, Germany). Urinary electrolytes (Na^+^, K^+^, Ca^2+^, Mg^2+^, Cl^−^) were measured by ion chromatography (Metrohm ion chromatograph, Herisau, Switzerland) and urinary creatinine was determined by the Jaffe method [33], [34].

### RNA extraction from kidney and reverse transcription

Snap-frozen kidneys were homogenized in RLT-Buffer (Qiagen, Basel, Switzerland) containing 2-mercaptoethanol (Sigma, Buchs, Switzerland) at a final concentration of 1%. Total RNA was extracted from 200 µl aliquots of each homogenized sample using the RNeasy Mini Kit (Qiagen, Basel, Switzerland) according to the manufacturer's instructions. Quantity and quality of total eluted RNA were assessed by spectrometry using the ND-1000 spectrophotometer (NanoDrop Technologies, DE, USA). Each RNA sample was diluted to 100 ng/µl, and 3 µl was used as a template for reverse transcription using the TaqMan Reverse Transcription Kit (Applied Biosystems, Foster City, CA, USA) according to manufacturer's protocol. Remaining RNA samples were stored at −80°C.

### Semiquantitative real-time RT-PCR

Semiquantitative real-time RT-PCR (qRT-PCR) was performed on the ABI PRISM 7700 Sequence Detection System (Applied Biosystems, Foster City, CA, USA). Primers for all genes of interest were designed using Primer Express software from Applied Biosystems and primers and probes were used as described previously [35], [36]. Probes were labeled with the reporter dye FAM at the 5′ end and the quencher dye TAMRA at the 3′ end (Microsynth, Balgach, Switzerland). The specificity of all primers was first tested in a standard PCR and always resulted in a single product of the expected size on 1% agarose gels (data not shown). Real-time PCR reactions were performed using the TaqMan Universal PCR Master Mix (Applied Biosystems, Foster City, CA, USA). Briefly, a 25 (20) µl PCR reaction volume was prepared using 3.5 (3) µl cDNA as template with sense and antisense primers (25 µM each), the labeled probed (5 µM), the Taqman Universal PCR Master Mix and RNase free water up to the final volume. Thermal cycles were set for denaturation at 95°C (10 min) followed by 40 cycles of denaturation at 95°C (15 s) and annealing/elongation at 60°C (1 min) with auto ramp time. All reactions were run in triplicates. To analyze the data, we set the threshold to 0.06 as this value had been determined to be in the linear range of the amplification curves for all mRNAs in all experimental runs. The expression of gene of interest was calculated in relation to hypoxanthine guanine phosphoribosyl transferase (HPRT). Relative expression ratios were calculated as R = 2^[Ct(HPRT)-Ct(test gene)]^, where Ct represents the cycle number at the threshold 0.06.

### Membrane preparation and western blot analysis

For total membrane preparation kidney cortex or medulla samples were homogenized in an ice-cold K-HEPES buffer (200 mM mannitol, 80 mM HEPES, 41 mM KOH, pH 7.5) containing a protease inhibitor mix (Complete Mini, Roche Diagnostics, Germany) at a final concentration of 1 tablet in a volume of 10 ml solution. Samples were centrifuged at 2000 rpm for 20 min at 4°C. Subsequently, the supernatant was transferred to a new tube and centrifuged at 41000 rpm for 1 h at 4°C. The resultant pellet was resuspended in K-HEPES buffer containing protease inhibitors. After measurement of the total protein concentration (Bio-Rad D_c_ Protein Assay; Bio-Rad, Hercules, CA, USA), 50 or 75 µg of crude membrane proteins were solubilized in Laemmli sample buffer, and SDS-PAGE was performed on 8–10% polyacrylamide gels. For immunoblotting, proteins were transferred electrophoretically to polyvinylidene difluoride membranes (Immobilon-P; Millipore, Bedford, MA, USA). After blocking with 5% milk powder in Tris-buffered saline/0.1% Tween-20 for 60 min, the blots were incubated with the respective primary antibodies (rabbit anti-pendrin 1∶5000 [25], rabbit anti-AE1 1∶10000 [33], diluted in 1%milk/TBS-T) and mouse monoclonal anti-β-actin antibody (42 kDa; Sigma, St. Louis, MO, USA) 1∶5000 either for 2 h at room temperature or overnight at 4°C. After washing and subsequent blocking, the membranes were incubated for 1 h at room temperature with the secondary antibody. Following secondary antibodies were used where appropriate: donkey anti-rabbit IgG-conjugated with horseradish peroxidase 1∶10000 (GE Healthcare, United Kingdom), sheep anti-mouse IgG-conjugated with horseradish peroxidase 1∶10000 (GE Healthcare, United Kingdom), and goat anti-rabbit or anti-mouse IgG-conjugated with alkaline phosphatase 1∶5000 (Promega, WI, USA). Antibody binding was detected with the Immobilon™ Western chemiluminescence kit (Millipore; Billerica, MA, USA) using the DIANA III-chemiluminescence detection system (Raytest; Straubenhardt, Germany). All images were analyzed using appropriate software (Advanced Image Data Analyzer, Raytest, Straubenhardt, Germany) to calculate the protein of interest/β-actin ratio.

### Immunohistochemistry

Mice were anesthetized as described above and subsequently perfused through the left ventricle with PBS followed by a paraformaldehyde-lysine-periodate fixative [37]. Kidneys were removed and fixed overnight at 4°C by immersion in paraformaldehyde-lysine-periodate. Kidneys were washed three times with PBS, and after cryoprotection with PBS/30% sucrose solution, thin coronal slices were cut, mounted on cryomolds, and frozen in liquid propane cooled with liquid nitrogen. Immunostaining was carried out as described previously [38]. Briefly, 5 µm thin sections were incubated with 1% SDS for 5 min, washed three times with PBS, and incubated with PBS containing 1% BSA for 15 min before application of the primary antibody. The primary antibodies (rabbit anti-AE1 1∶10000 [33], guinea pig anti-pendrin 1∶2000 [25], mouse anti-calbindin 1∶40000 (SWANT, Bellinzona, Switzerland) and goat anti-AQP2 1∶400 (Chemicon (Millipore), Zug, Switzerland) were diluted in PBS/0.02% Na-azide and applied either for 75 min at room temperature or overnight at 4°C. Sections were then washed twice for 5 min with high-NaCl-PBS (PBS+ 18 g NaCl/l), once with PBS, and incubated with the secondary antibodies (donkey anti-rabbit Alexa 594 1∶1000, donkey anti-guinea pig Cy2 1∶400, donkey anti-mouse Cy5 1∶500, donkey anti-goat Cy5 1∶500 (Invitrogen, Basel, Switzerland) and DAPI 1∶500 (Sigma)) at the given dilutions for 1 h at room temperature. Sections were again washed twice with high-NaCl-PBS and once with PBS before being mounted with Glycergel (DakoCytomation, Glostrup, Denmark). Sections were viewed using a Leica DFC490 charged-coupled device camera attached to a Leica DM 6000 fluorescence microscope (Leica, Wetzlar, Germany) using equivalent camera parameters for kidneys sections stained with the same primary antibody. Pictures were processed using Imaris. Cell counting for the different subtypes of intercalated cells was performed as described previously [39].

### Statistical Analysis

Results are expressed as means ± SEM. All data were tested for significance using ANOVA and unpaired Student's t-test where appropriate. Results with P<0.05 were considered as significant.

## Results

**Figure 1:**
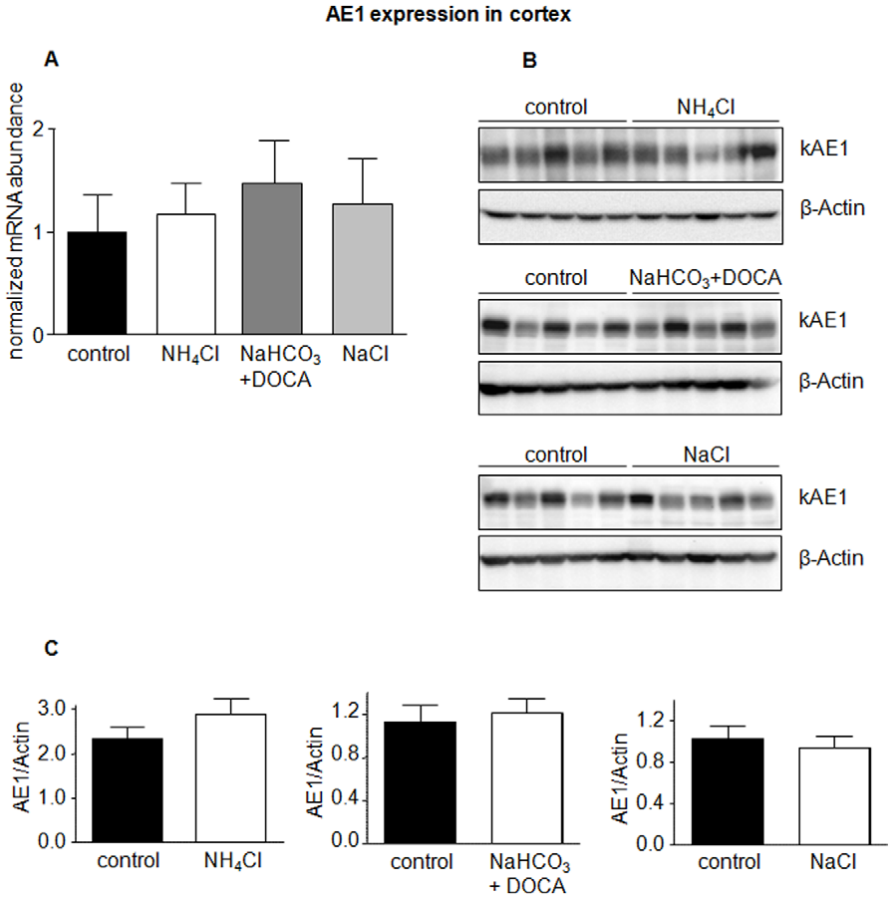
AE1 expression is not regulated in kidney cortex. The mRNA and protein abundance of the type A intercalated cell specific Cl^−^/HCO_3_^−^ exchanger AE1 was measured by real-time RT-PCR (**A**) and immunoblotting (**B**) in kidney cortex. (**C**) Bar graphs summarizing data from immunoblotting. All data were normalized against β-actin obtained on the same immunoblotting membrane after stripping. Data are given as normalized mean ± SE; *n* = 5–7 animals/group.

**Figure 2:**
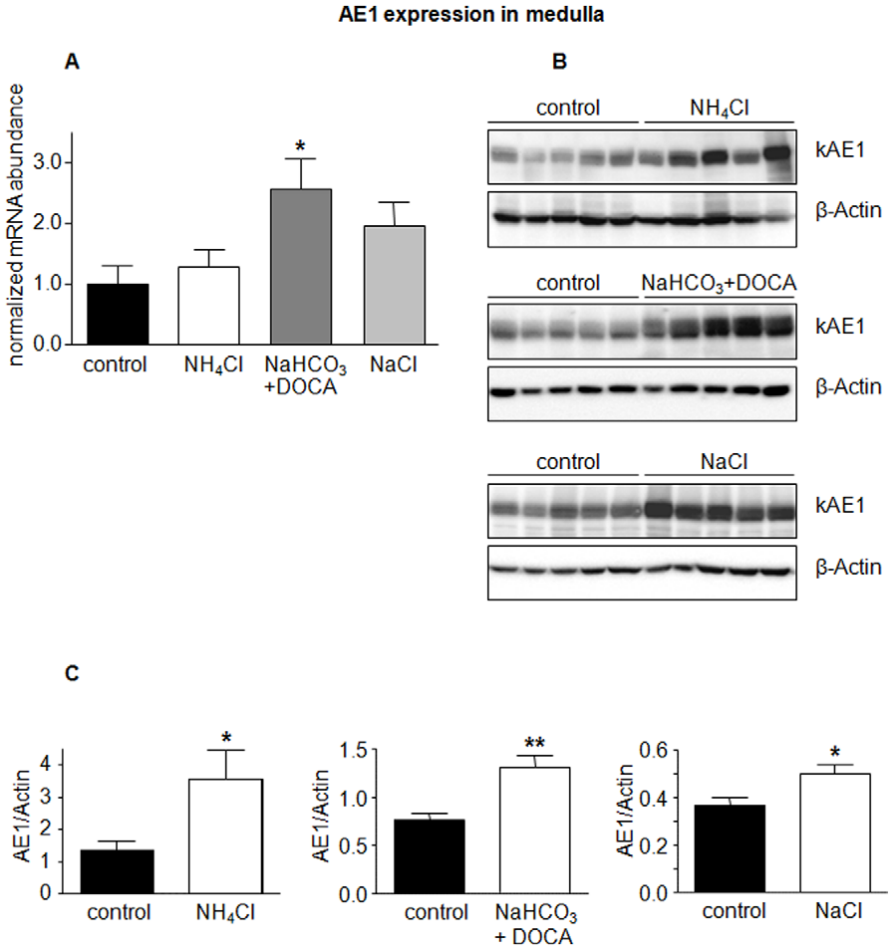
Regulated expression of AE1 in kidney medulla. mRNA and protein abundance of the type A intercalated cell specific Cl^−^/HCO_3_^−^ exchanger AE1 was measured by real-time RT-PCR (**A**) and immunoblotting (**B**) in kidney medulla. (**C**) Bar graphs summarizing data from immunoblotting. All data were normalized against β-actin obtained on the same immunoblotting membrane after stripping. Data are given as normalized mean ± SE; *n* = 5–7 animals/group. *p≤0.05, **p≤0.01.

The anion exchangers AE1 and pendrin have been shown to be regulated during acidosis and alkalosis [25], [40], [41], [42], [43]. In a first series of experiments mice received NH_4_Cl to induce metabolic acidosis, a combination of NaHCO_3_ and the aldosterone analogue deoxycorticosterone acetate (DOCA) to induce alkalosis, and NaCl to test for effects of sodium and chloride. Urine analysis confirmed the expected effects such as lower urine pH and increased ammonium excretion in NH_4_Cl-loaded mice or increased sodium excretion in mice treated with NaCl or NaHCO_3_ (table 1). Semiquantitative RT-PCR did not detect any difference in AE1 mRNA levels in kidney cortex (Figure 1A) but showed significantly elevated AE1 mRNA in kidney medulla in mice receiving NaHCO_3_ and DOCA (Figure 2A). Similarly, AE1 protein abundance in kidney cortex was not affected by treatments (Figure 1B,C), whereas NH_4_Cl and NaHCO_3_ in combination with DOCA significantly stimulated AE1 protein expression in kidney medulla (Figure 2B,C). Treatment with NaCl tended to increase AE1 mRNA and significantly enhanced protein in medulla (Figure 2C).

**Table 1:** Summary of urine data from mice receiving different treatments.

	pH	Volume/BW (g/g)	PO_4_/creatinine (mM/mg/dl)	NH_4_^+^/creatinine (mM/mg/dl)	Na^+^/creatinine (mM/mg/dl)	K^+^/creatinine (mM/mg/dl)	Cl^−^/creatinine (mM/mg/dl)
Control 1	6.19±0.08	0.04±0.01	1.00±0.15	0.77±0.19	1.97±0.21	4.66±0.28	4.56±0.51
NH_4_Cl	5.48±0.02***	0.04±0.01	0.61±0.17	6.54±0.81**	2.57±0.33	5.54±0.51	11.68±1.61**
NaHCO_3_+DOCA	8.48±0.15***	0.10±0.03	0.62±0.21	0.08±0.03***	15.28±2.01***	3.83±0.52	3.84±0.36
NaCl	6.30±0.14	0.09±0.02	0.79±0.11	0.25±0.02**	11.34±0.89***	3.93±0.48	17.08±2.18***
Control 2	6.28±0.08	0.04±0.01	0.93±0.14	0.42±0.02	2.23±0.16	6.30±0.44	4.44±0.39
DOCA	6.52±0.02*	0.05±0.01	1.10±0.14	0.77±0.06***	2.88±0.22*	7.72±0.31*	5.80±0.17**
Control 3	6.34±0.07	0.09±0.01	1.41±0.13	0.42±0.03	2.71±0.34	9.34±1.11	5.17±0.65
NaHCO_3_	8.29±0.18***	0.05±0.01	0.93±0.10*	0.06±0.01***	13.12±0.47***	6.47±0.40	3.87±0.30
Control 4	6.00±0.13	0.05±0.00	0.94±0.06	0.79±0.20	1.34±0.05	5.19±0.33	3.59±0.27
KCl	5.75±0.17	0.12±0.03*	1.36±0.12*	0.66±0.16	3.15±0.28***	29.70±4.07***	24.96±4.97**
KHCO_3_	8.11±0.09***	0.10±0.01**	1.07±0.16	0.23±0.13*	1.97±0.34	20.21±2.48***	4.56±0.60

All animals were treated for 7 days and placed into metabolic cages for the final 3 days. Urine analysis was performed from samples collected under mineral oil over the last 24 hrs. Values are means ± SE; *n* = 5–7/group. Shown is a summary of urinary data from all different treatment groups and the corresponding controls. BW, body weight, crea creatinine.

* *p*<0.05,

** *p<*0.01,

*** *p*<0.001.

**Figure 3:**
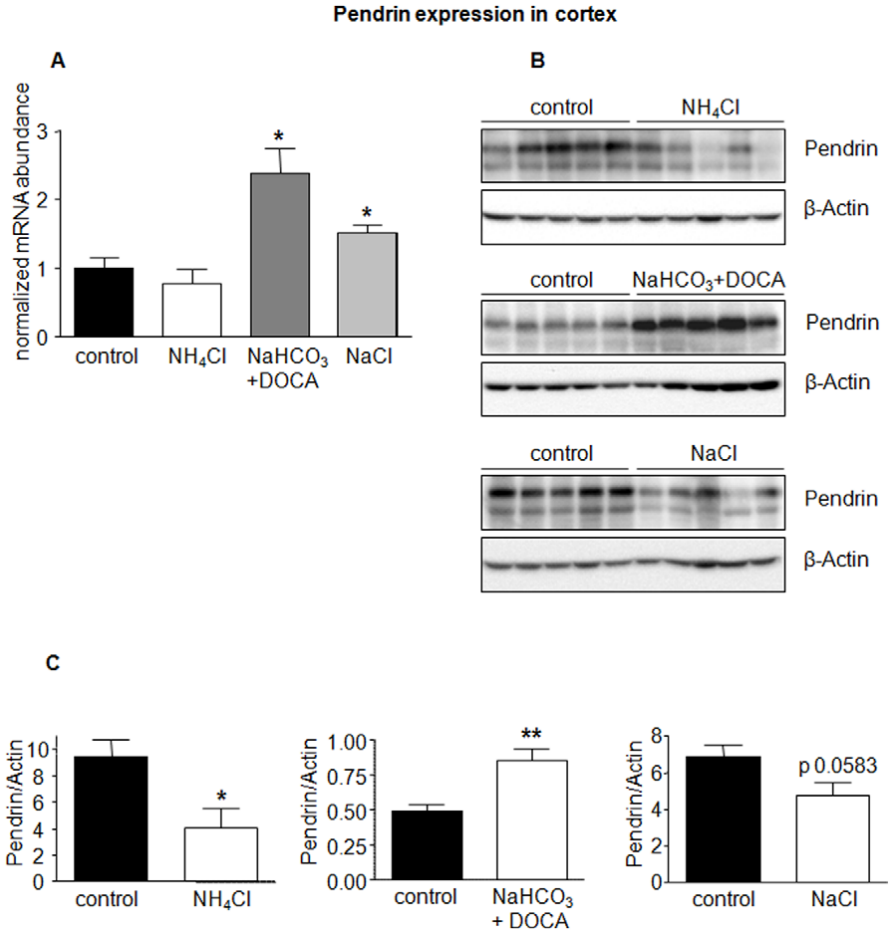
Pendrin expression in kidney cortex regulated by various treatments. The mRNA and protein abundance of the type B intercalated cell specific Cl^−^/HCO_3_^−^ exchanger pendrin was measured by real-time RT-PCR (**A**) and immunoblotting (**B**) in kidney cortex. (**C**) Bar graphs summarizing data from immunoblotting. All data were normalized against β-actin obtained on the same immunoblotting membrane after stripping. Data are given as normalized mean ± SE; *n* = 5–7 animals/group. *p≤0.05, **p≤0.01.

In the same mice, treatment with NaHCO_3_/DOCA or NaCl alone enhanced pendrin mRNA significantly; the combination of NaHCO_3_ and DOCA being more effective than NaCl (Figure 3A). On the protein level, NH_4_Cl significantly reduced pendrin abundance, whereas NaHCO_3_/DOCA increased pendrin expression (Figure 3B,C). NaCl reduced pendrin expression, which reached almost statistical significance (p = 0.058). Pendrin expression was examined only in cortical preparations since pendrin is mostly expressed in the cortical segments of the collecting system (e.g. the late distal convoluted tubule, CNT and CCD).

**Figure 4:**
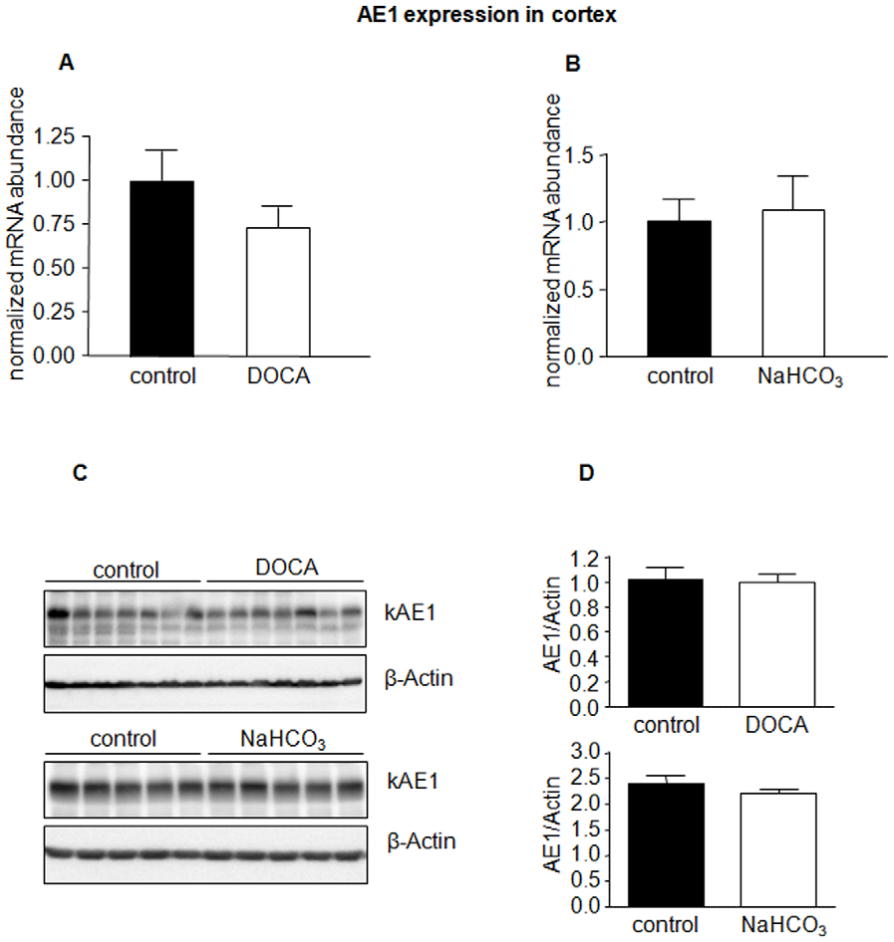
DOCA or NaHCO_3_ alone do not alter AE1 expression in kidney cortex. The mRNA and protein abundance of AE1 in kidney cortex from mice treated with NaHCO_3_ or DOCA alone was measured by real-time RT-PCR (**A, B**) and immunoblotting (**C**) in the kidney cortex. (**D**) Bar graphs summarizing data from immunoblotting. All data were normalized against β-actin obtained on the same immunoblotting membrane after stripping. Data are given as normalized mean ± SE; *n* = 5–7 animals/group.

**Figure 5:**
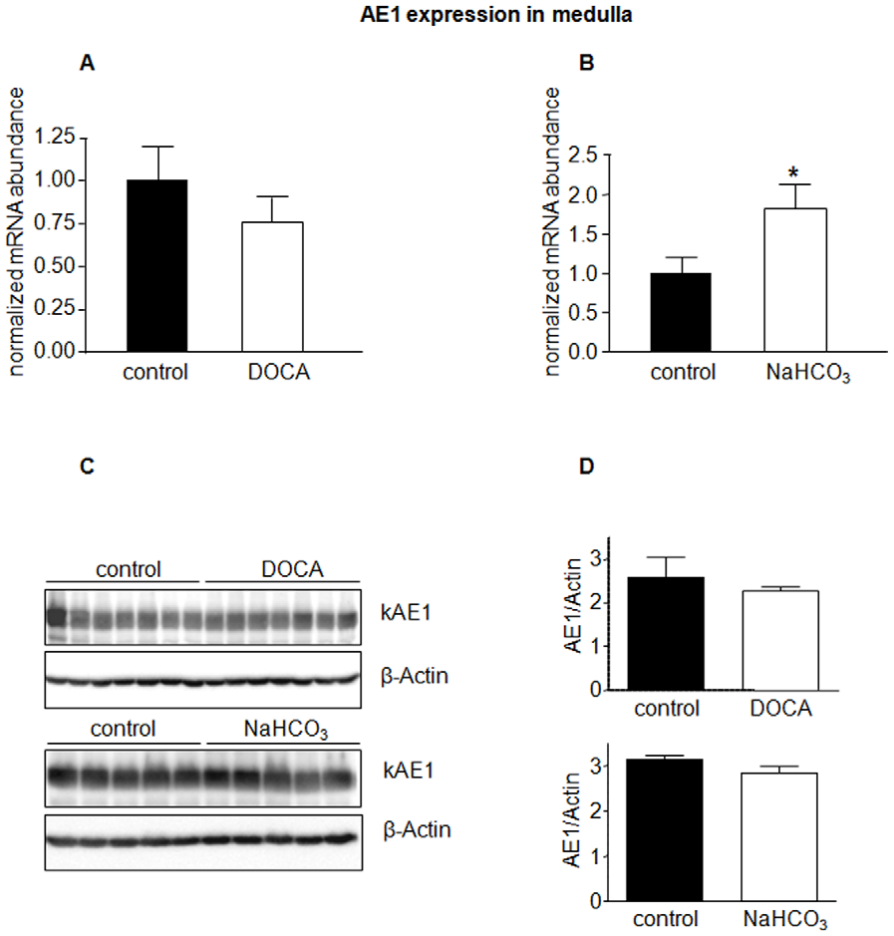
NaHCO_3_ increases mRNA expression of AE1 in kidney medulla. The mRNA and protein abundance of AE1 in kidney medulla from mice treated with NaHCO_3_ or DOCA alone was measured by real-time RT-PCR (**A,B**) and immunoblotting (**C**) in kidney medulla. (**D**) Bar graphs summarizing data from immunoblotting. All data were normalized against β-actin. Data are given as normalized mean ± SE; *n* = 5–7 animals/group. *p≤0.05,

**Figure 6:**
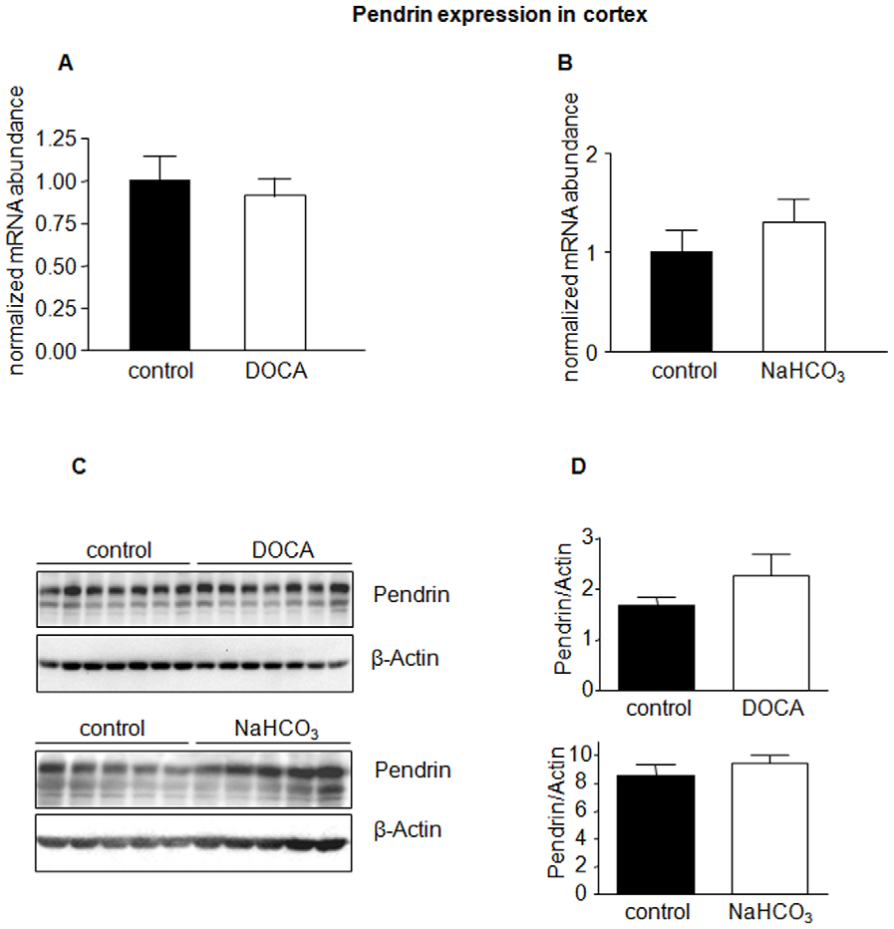
Pendrin expression is not regulated by DOCA or NaHCO_3_ alone. Pendrin mRNA and protein abundance in kidney cortex from mice treated with NaHCO_3_ or DOCA alone for 7 days was measured by real-time RT-PCR (**A,B**) and immunoblotting (**C**). All membranes were stripped and reprobed for β-actin. (**D**) Bar graphs summarizing data from immunoblotting. All data were normalized against β-actin. Data are given as normalized mean ± SE; *n* = 5–7 animals/group.

In a next series of experiments, we examined if the effects of NaHCO_3_/DOCA were induced by either NaHCO_3_ or DOCA alone, or by combined treatment. Mice received therefore either NaHCO_3_ or DOCA alone. The efficacy of treatments was confirmed by urine analysis (table 1). NaHCO_3_ or DOCA alone had no effect on AE1 mRNA or protein expression in kidney cortex (Figure 4), whereas in medulla NaHCO_3_ increased AE1 mRNA but not protein abundance (Figure 5). DOCA was without effect on mRNA and protein expression in cortex and medulla (Figure 5). Moreover, pendrin mRNA and protein abundance were unaffected by either of the treatments (Figure 6).

**Figure 7:**
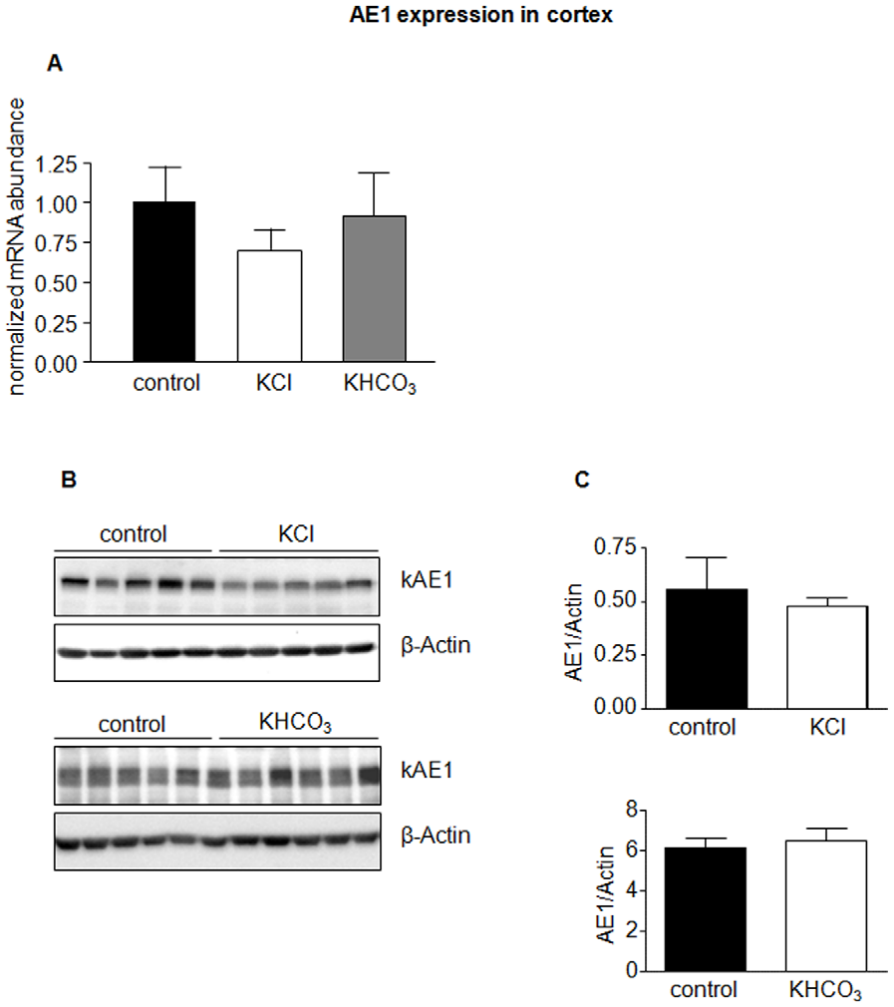
AE1 in cortex is not affected by dietary KCl and KHCO_3_. AE1 mRNA and protein abundance in kidney cortex from mice treated with KCl or KHCO_3_ for 7 days was measured by real-time RT-PCR(**A**) and immunoblotting (**B**). All membranes were stripped and reprobed for β-actin. (**C**) Bar graphs summarizing data from immunoblotting. All data were normalized against β-actin. Data are given as normalized mean ± SE; *n* = 5–7 animals/group.

**Figure 8:**
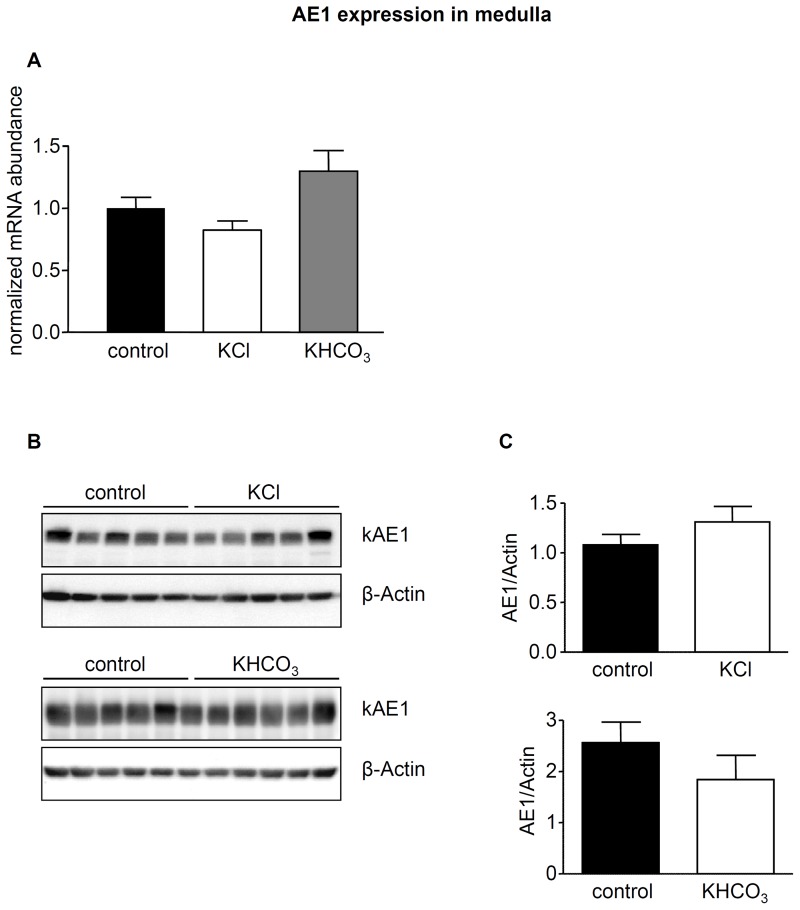
Medullay AE1 is not regulated by KCl and KHCO_3_. AE1 mRNA and protein abundance in kidney medulla from mice treated with KCl or KHCO_3_ for 7 days were measured by real-time RT-PCR (**A**) and immunoblotting(**B**). All membranes were stripped and reprobed for β-actin. (**C**) Bar graphs summarizing data from immunoblotting. All data were normalized against β-actin. Data are given as normalized mean ± SE; *n* = 5–7 animals/group.

**Figure 9:**
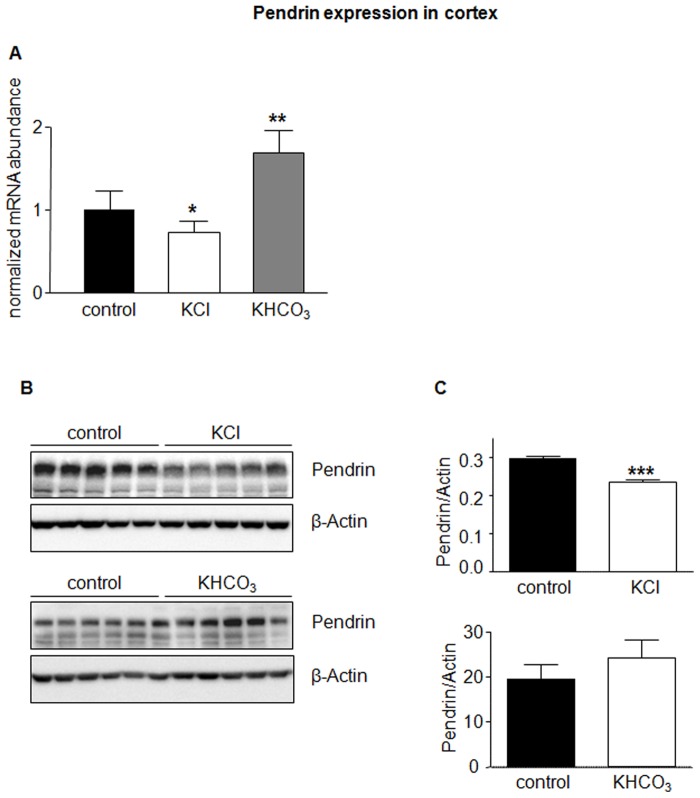
KCl and KHCO_3_ regulate pendrin expression. Pendrin mRNA and protein abundance in kidney cortex from mice treated with KCl or KHCO_3_ for 7 days were examined by real-time RT-PCR (**A**) and immunoblotting (**B**). All membranes were stripped and reprobed for β-actin. (**C**) Bar graphs summarizing data from immunoblotting. All data were normalized against β-actin. Data are given as normalized mean ± SE; *n* = 5–7 animals/group. *p≤0.05, **p≤0.01, ***p≤0.001.

In a third series of experiments, we tested whether chloride or bicarbonate had different effects when combined with potassium instead of sodium as counter cation. Mice received either KCl or KHCO_3_ for 7 days and subsequently urine was analyzed to confirm effectiveness of treatment (table 1). Both diets were without significant effect on AE1 mRNA and protein abundance in kidney cortex and medulla (Figures 7 and 8). In contrast, KCl reduced pendrin mRNA and protein significantly (Figure 9). KHCO_3_ treatment enhanced pendrin mRNA abundance approximately two-fold but pendrin protein expression remained unchanged (Figure 9).

In a last series of experiments we examined the effects of NH_4_Cl, KCl, and NaHCO_3_ on the relative abundance and distribution of AE1 and pendrin expressing intercalated cell subtypes in kidney cortex and medulla. Detailed counting of cells in the different segments of the collecting system revealed significant changes only for animals receiving NH_4_Cl (table 2).

**Table 2:** Relative abundance of the various cells types along the collecting system in kidneys from control mice and mice treated with KCl, NH_4_Cl, or NaHCO_3_.

	AE1 pos, Pendrin neg, AQP2 neg, %	Pendrin pos, AE1 neg, AQP2 neg, %	AQP2 pos, AE1 neg, Pendrin neg, %
**CNT**			
Control	13.1±0.7 (441)	30.5±0.8 (1014)	54.8±0.8 (1720)
KCl	13.6±0.6 (537)	28.6±0.6 (1138)	57.4±0.7 (2369)
NH_4_Cl	17.1±0.4 (1264)***	25.0±0.5 (1821)***	57.6±0.5 (4166)
NaHCO_3_	15.1±0.6 (648)	30.2±0.6 (1383)	54.6±0.7 (2504)
**CCD**			
Control	16.6±1.0 (136)	19.7±1.0 (162)	63.7±1.3 (521)
KCl	13.5±1.4 (91)	20.2±1.6 (119)	65.6±2.2 (440)
NH_4_Cl	16.5±1.4 (167)	19.5±1.1 (204)	63.9±1.3 (654)
NaHCO_3_	14.8±1.5 (148)	21.7±1.4 (233)	63.3±1.7 (668)
**OMCD**			
Control	26.9±3.1 (112)	6.6±2.1 (13)	65.6±2.4 (256)
KCl	29.2±2.4 (153)	2.6±1.7 (7)	67.3±2.2 (364)
NH_4_Cl	33.8±1.6 (335)*	3.0±1.4 (9)	63.2±1.5 (611)*
NaHCO_3_	31.4±1.7 (244)	2.2±1.5 (7)	66.3±1.4 (484)
**IMCD_i_**			
Control	22.2±1.4 (263)	0±0 (0)	77.5±1.5 (1012)
KCl	19.6±3.0 (118)	0±0 (0)	80.4±3.0 (626)
NH_4_Cl	21.8±2.8 (294)	0±0 (0)	78.2±2.8 (1119)
NaHCO_3_	21.4±2.6 (350)	0±0 (0)	78.6±2.6 (1515)

Values are means ± SE, with the number of cells counted in parentheses. Shown is a summary of cell counts in the different segments of the collecting system, 5 animals in each group were analyzed, and cells were counted. CNT, connecting tubule; CCD, cortical collecting duct; OMCD, outer medullary collecting duct; IMCD_i_, initial segment of the inner medullary collecting duct. Significant difference between control and treatment groups:

* p<0.05,

** p<0.01,

*** p<0.001.

Kidney sections from animals receiving sucrose (control), NH_4_Cl, KCl, or NaHCO_3_ for 7 days were stained for AE1, pendrin, AQP2/calbindin D28k, and DAPI and cells counted for the various markers and distinct segments. The total number of cells counted is given in brackets, for each condition n = 5 animals were counted.

**Figure 10:**
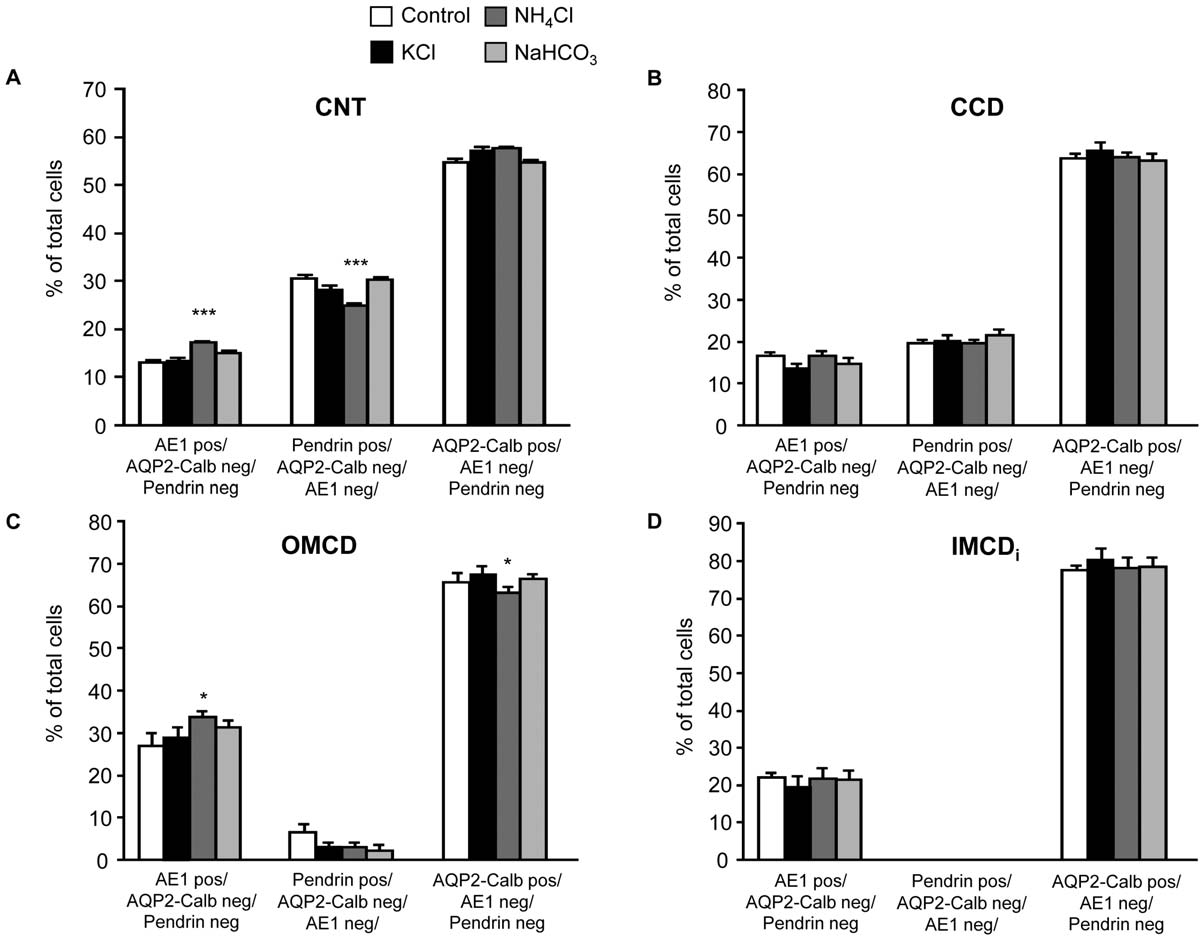
Remodeling of the collecting duct. Kidneys from mice left untreated (sucrose, control) or receiving KCl, NaHCO_3_ or NH_4_Cl for 7 days were sectioned and stained for specific cell markers (AE1 for type A intercalated cells, pendrin for non-type A intercalated cells, and calbindin D28k and AQP2 for principal cells). The relative number of cells expressing these markers was counted for the different segments of the collecting system. CNT connecting tubule, CCD cortical collecting duct, OMCD outer medullary collecting duct, _i_IMCD initial inner medullary collecting duct, *p≤0.05, ***p≤0.001.

**Figure 11:**
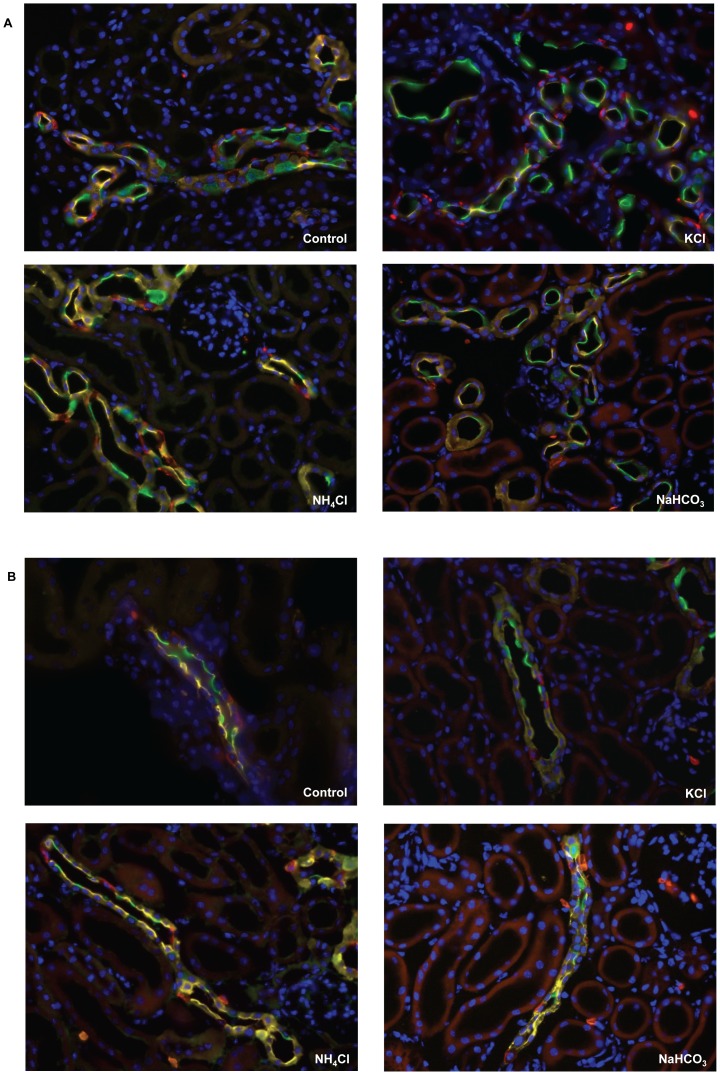
Immunolocalization of intercalated cell subtypes in mouse kidney under different dietary treatments. Mice were left untreated (sucrose, control) or received NH_4_Cl, NaHCO_3_ or KCl in their drinking water for 7 days. Immunohistochemistry was performed to identify type A intercalated cells with AE1 (red), non-type A intercalated cells with pendrin (green), principal cells with calbindin D28k/AQP2 (yellow), and nuclei (DAPI, blue). Representative pictures are shown from CNT (**A**) and CCD (**B**). Original magnification 400×.

In these animals, a significant increase of AE1 expressing cells in the connecting tubule (CNT) and outer medullary collecting duct (OMCD) was noted that was paralleled by a decrease of pendrin positive cells in the CNT and aquaporin 2 (AQP2) positive cells in the OMCD, respectively. All other small changes did not reach statistical significance (Figures 10 and 11, table 2).

## Discussion

Intercalated cells have been traditionally viewed as being important for the control of systemic acid-base homeostasis by excreting acid or base equivalents [15]. However, in more recent years several links to the renal control of salt and electrolyte handling have emerged. Secretion of potassium by flow-activated maxiK channels expressed in intercalated cells has been observed [44], [45]. The identification of pendrin as major apical anion transporter in non-type A intercalated cells has linked this cell type to the reabsorption of chloride and its impact on blood pressure regulation [8], [22], [28], [46]. More recent work by Eladari and colleagues identified a Na^+^-driven chloride/bicarbonate transporter, Slc4a8, that may in conjunction with pendrin mediate electroneutral NaCl reabsorption. Interestingly, the activity of Slc4a8 is also thiazide sensitive [18].

Here we demonstrate that expression of AE1 and pendrin is sensitive to dietary electrolyte intake and the aldosterone analogue DOCA. Several observations are important. First, regulation on mRNA and protein level do not always go in parallel, sometimes even in opposite directions calling for caution in drawing conclusions about protein expression from mRNA data. Of note, we did not determine transport activities in the present study, thus, our data on protein and cell abundances do not necessarily translate into changes in activity. However, the data on blood and urine composition indicate parallel changes suggesting that protein abundance and function may be closely linked. Second, AE1 mRNA and protein expression in cortex was unchanged in all groups whereas protein expression in kidney medulla was strongly regulated by NH_4_Cl, NaHCO_3_/DOCA, and NaCl. Third, regulation of AE1 in the medulla was only observed if NaHCO_3_ was combined with DOCA, but not in the groups receiving DOCA, NaHCO_3_ or KHCO_3_ alone. These data therefore suggest, that alkali treatment, sodium, potassium or DOCA alone do not affect AE1 protein expression. DOCA, or its natural analogue aldosterone, may therefore have a permissive effect on AE1 expression when combined with alkalosis. This effect may be of clinical relevance in patients with stimulated aldosterone levels and alkalosis as seen in volume contracted patients and would consequently contribute to maintenance of alkalosis or even enhance it. The increase in AE1 mRNA in medulla in NaHCO_3_ treated animals did not translate into more protein. NH_4_Cl increased AE1 protein but not mRNA abundance in medulla. Consistently, we did not detect significant AE1 mRNA changes in a genome-wide transcriptome analysis of mouse kidney during acidosis in earlier experiments [36]. Though, in rat kidney, AE1 is regulated by NH_4_Cl both on mRNA and protein level [43]. The reason for this species difference is presently unknown.

Our study further showed that AE1 is not regulated by dietary chloride intake, since KCl failed to change AE1 abundance. On the other hand, NaCl tended to increase AE1 mRNA and stimulated protein expression in kidney medulla. Since NaHCO_3_ alone had no effect on AE1 mRNA or protein expression, we reason that sodium delivery alone does not regulate AE1. NaCl availability has been shown to enhance the kidneys ability to excrete acid [47]. Increased expression of AE1 under these conditions could contribute to removal of acid and generation of new bicarbonate.

Pendrin regulation by aldosterone and various electrolytes has been studied in some detail [10], [22], [25], [26], [27], [28], [40], [41], [46]. Our results here confirm regulation of pendrin by chloride availability. Dietary supplementation with NH_4_Cl, NaCl or KCl increased urinary chloride excretion and reduced pendrin protein abundance. Interestingly, these treatments had different effects on pendrin mRNA expression. KCl reduced mRNA levels, whereas NaCl increased it, and NH_4_Cl had no effect. Thus, chloride may not directly affect pendrin mRNA but additional factors must be operative that regulate pendrin transcription or mRNA stability.

Mice were treated with bicarbonate in combination with either sodium or potassium as counter cations and in the absence or presence of DOCA, respectively. Bicarbonate alone did not regulate pendrin mRNA or protein over a period of 7 days since NaHCO_3_ alone did affect neither mRNA nor protein, whereas KHCO_3_ or NaHCO_3_ in combination with DOCA increased mRNA abundance. Only the combination of NaHCO_3_/DOCA increased also pendrin protein expression. DOCA alone was without effect on mRNA and protein. Thus, the combination of aldosterone and bicarbonate is a potent stimulus to enhance pendrin expression. In the case of KHCO_3_, the potassium load may increase serum aldosterone levels but to a lesser extent than in animals with DOCA administration. Our results are not in agreement with two previous reports. Verlander *et al.* reported that DOCP alone enhanced pendrin expression but mice had received additional NaCl supplements and only one application of DOCP seven days before organ collection [22]. We speculate that the additional NaCl load and the different application regimen may alter the response to the aldosterone analogue. Alternatively, the effect of DOCA or its analogues may be time-dependent and transient and only detectable after short treatments but not after 7 days [48]. A second study addressed regulation of human pendrin transcription by aldosterone using HEK293 cells as cellular model. These cells are derived from human embryonic kidney and are not representative for collecting duct intercalated cells. In these cells, aldosterone suppressed pendrin transcription [29], while our results suggest that DOCA alone has no effect on pendrin mRNA abundance and that a second stimulus, i.e. bicarbonate, is needed to enhance pendrin mRNA. Thus, transcriptional regulation of pendrin differs between cell models and in vivo [49], which may be the result of the interaction of several factors affecting the collecting duct, and not all transcriptional changes translate into altered protein abundance demonstrating an additional level of control.

Remodeling of the collecting duct occurs in response to changes in electrolyte and/or acid-base status [25], [38], [40], [50], [51], [52], [53], [54], [55], [56], [57], [58], [59]. Induction of acidosis with NH_4_Cl has been shown to increase the relative number of type A intercalated cells in various rat and mouse models. AE1 protein expression increased during NH_4_Cl only in the medulla but not in cortex. In contrast, we found a higher relative number of AE1 expressing cells in both cortex and medulla during NH_4_Cl-loading suggesting that in the cortex the abundance of AE1 per cell may be stimulated. Pendrin abundance in the cortex was reduced during NH_4_Cl loading and the relative number of pendrin positive cells decreased in parallel. However in KCl-loaded animals, pendrin protein abundance decreased without a significant chance in the relative number of pendrin expressing cells consistent with a possible reduction of pendrin expression per cell. In both cases, the cellular mechanisms responsible for sensing changes in acid-base status or chloride concentrations are unknown. Also the mechanisms triggering the reduction in protein expression or cell number have not been identified to date. Several mechanisms have been suggested to be involved in the remodeling of the collecting duct in response to NH_4_Cl-induced acidosis including proliferation of type A intercalated cells and hensin-mediated interconversion of type A and non-A intercalated cells [56], [59], [60].

In summary, the abundance of the two major anion exchangers AE1 and pendrin expressed in the different subtypes of intercalated cells is heavily regulated by electrolyte intake, acid-base status, and the aldosterone analogue. In some instances the presence of two combined stimuli is required for regulation. Apparently, regulation involves changes in protein expression per cell as well as changes in the relative abundance of the two major intercalated cell subtypes. The adaptive regulation of the type A intercalated cell anion exchanger AE1 mainly follows changes in acid-base status whereas regulation of pendrin expression paralleled mostly chloride intake and acid-base status. Our data demonstrate differential regulation of these transporters and the two intercalated subtypes. The altered expression may support the renal response to changes in acid-base status and electrolyte intake but may also contribute in some instances to the maintenance of electrolyte and acid-base disturbances.
